# Supplementary material for: Accuracy of hepatitis B disease surveillance, Gannan prefecture, Gansu province, China; 2017
Source: PLoS One. 2022 Sep 19;17(9):e0274798. doi: 10.1371/journal.pone.0274798 (PMC9484802; doi:10.1371/journal.pone.0274798)
Supplement: Attachment — Submitted filename: 2022-8-1 Rsponse to editor and reviewers(1).docx [file pone.0274798.s002.docx]

Dear editor,

Thanks for your and the reviewers’ valuable comments and recommendations. We had re-checked all the references in the article and replaced reference number 1 with the latest one. We corrected some authors’ name spelling and the journal spelling according to PLOS journal demand. The URL for some references were updated, and some missing literature DOI addresses were added.

We deleted the supporting information part because it was the same as the tables in the main manuscript.

For the second reviewer‘s comments, we recommended the hospitals to conduct education campaign about HBV diagnosis and reporting criteria in the discussion part in the main manuscript.

Thanks again for all your comments.

Best regards,

Lijie Zhang, China CDC

Correspondent author
